# Wearable Devices for Remote Monitoring of Chronic Diseases: Systematic Review

**DOI:** 10.2196/74071

**Published:** 2026-02-11

**Authors:** Masresha Derese Tegegne, Sharareh Rostam Niakan Kalhori, Paulo Haas, Viktor MG Sobotta, Joana Warnecke, Thomas M Deserno

**Affiliations:** 1 Peter L. Reichertz Institute for Medical Informatics of TU Braunschweig and Hannover Medical School, Mühlenpfordtstraße 23, Braunschweig, 38106, Germany, +49 531 391 9510; 2 Department of Health Information Management and Medical Informatics, School of Allied Medical Sciences, Tehran University of Medical Sciences, Tehran, Iran

**Keywords:** wearable, sensor, health parameter, vital sign, body location, medical application, standardized terminology

## Abstract

**Background:**

Wearable devices enable the remote collection of health parameters, supporting the outpatient care plans recommended by the World Health Organization to manage chronic diseases. While disease-specific monitoring is accurate, a comprehensive analysis of wearables across various chronic diseases helps to standardize remote patient monitoring systems.

**Objective:**

This review aimed to identify wearables for remote monitoring of chronic diseases, focusing on (1) wearable devices, (2) sensor types, (3) health parameters, (4) body locations, and (5) medical applications.

**Methods:**

We developed a search strategy and conducted searches across three databases: PubMed, Web of Science, and Scopus. After reviewing 1160 articles, we selected 61 that addressed cardiovascular, cancer, neurological, metabolic, respiratory, and other diseases. We created a data analysis method based on our 5 objectives to organize the articles for a comprehensive analysis.

**Results:**

From the 61 articles, 39 (64%) used wearable bands such as smartwatches, wristbands, armbands, and straps to monitor chronic diseases. Wearable devices commonly included various sensor types, such as accelerometers (n=39, 64%), photoplethysmographic sensors (n=18, 30%), biopotential meters (n=17, 28%), pressure meters (n=11, 18%), and thermometers (n=9, 15%). These sensors collected diverse health parameters, including acceleration (n=39, 64%), heart rate (n=24, 39%), body temperature (n=9, 15%), blood pressure (n=8, 13%), and peripheral oxygen saturation (n=7, 11%). Common sensor body locations were the wrist, followed by the upper arm and the chest. The medical applications of wearable devices were neurological (n=21, 34%) and cardiovascular diseases (n=15, 25%). Additionally, researchers applied wearable devices for wellness and lifestyle monitoring (n=39, 64%), mainly for activity (n=39, 100%) and sleep (n=10, 26%).

**Conclusions:**

This review underscores that wearable devices primarily function as bands, commonly worn on the wrist, to monitor chronic diseases. These devices collect data on acceleration, heart rate, body temperature, blood pressure, and peripheral oxygen saturation, with a focus on neurological and cardiovascular diseases. Our findings provide a foundational road map for designing generalized remote patient monitoring systems to manage multimorbidity and support standardized terminology for interoperability across digital health systems. To translate this into practice, we recommend that future research prioritize pragmatic clinical trials with medically certified devices.

## Introduction

### Background

According to the World Health Organization (WHO), chronic diseases are long-lasting, noninfectious, and progressively worse over time [1]. These include cardiovascular diseases, neurological disorders, cancer, respiratory diseases, and metabolic disorders, which collectively account for 74% of annual global mortality and make them the leading cause of death worldwide [1,2]. The burden of chronic diseases extends beyond health, severely straining health care resources. The Centers for Disease Control and Prevention reports that 90% of total health care expenditures in the United States address individuals with chronic diseases [3]. This expenditure encompasses ongoing treatments, regular medical consultations, and other health care–related costs [4].

Owing to their long-lasting nature and the ongoing commitment to curative health services, health care professionals mostly manage chronic diseases on an outpatient basis [5]. However, preventable factors related to these chronic diseases lead to sudden death [6]. The WHO prioritizes individuals with chronic diseases and underscores the need to develop efficient outpatient treatment strategies [7]. Health care providers achieve this by remotely monitoring health parameters [8,9]. Compared to inpatient health care delivery, remote patient monitoring (RPM) systems have become a promising option for managing chronic diseases [10,11]. RPM systems support early detection by real-time monitoring of health and well-being [12] using wearable devices that integrate 1 or more sensors to collect relevant health parameters remotely [13-15]. This reduces clinic visits and conserves time and health care resources [16].

Wearable-based RPM systems rely on custom-built sensors to gather and analyze biometric and physiological data [17]. Contemporary devices such as smartwatches and fitness trackers have several sensors [18] and often pair with mobile apps. Integrating these wearable devices with cloud platforms facilitates efficient data storage and easy access for health care providers. Examples of cloud platforms include Apple HealthKit [19], Google Fit [20], Microsoft Azure Health Data Services [21], Amazon AWS HealthLake [22], and Biofourmis [23]. Such technologies offer substantial potential for clinical trials, fostering research advancements and improving patient care.

Current articles on wearable devices highlight their potential in managing chronic diseases. Various sensors, such as accelerometers, gyroscopes, magnetometers, biopotential meters, photoplethysmographic (PPG) sensors, and thermometers, monitor health parameters remotely. These include activity, electrocardiography (ECG), electroencephalography, electromyography, heartbeats, heart rate, sleep patterns, and body temperature [24-31].

Currently, wearable devices play a key role in monitoring specific chronic diseases, enhancing the effectiveness of RPM by focusing on disease-specific health parameters. For instance, continuous glucose monitors track glucose levels in diabetes management [32], biopotential meters monitor heart rhythms in various conditions [33], and inertial measurement units (IMU) evaluate body movements for diagnosing stroke and neurodegenerative diseases [34,35].

Previous review articles on wearable devices for remote monitoring of chronic diseases often focus on a single condition, a specific population, or a limited clinical setting [36,37]. This narrow focus creates a knowledge gap in understanding their applicability across a broader range of chronic diseases. The increasing variability of wearable devices and stand-alone RPM systems presents substantial challenges for interoperability with digital health systems [38,39], including electronic health record systems [40]. These challenges become even more pressing in the context of rising rates of multimorbidity [41], where patients require integrated monitoring solutions that can simultaneously track multiple conditions. This systematic review addresses these gaps by applying a five-category standardized terminology framework to analyze wearable devices used for remote monitoring of chronic diseases.

### Objectives

This systematic review aimed to explore wearable devices and sensor types for remote monitoring of chronic diseases. Specifically, we answered the following research questions:

1. Which wearable devices are used to monitor chronic diseases?
2. Which sensor types are integrated into these wearable devices?
3. Which health parameters do these sensors collect?
4. Where are these sensors located on the human body?
5. Which medical applications are supported by the wearable devices?

## Methods

### Overview

This systematic review’s design and reporting follow the PRISMA (Preferred Reporting Items for Systematic Reviews and Meta-Analyses) guidelines (Checklist 1) [42]. We registered the protocol in PROSPERO (CRD42023460873). Although we specified Quality Assessment Tool for Diverse Designs for quality appraisal in the registered protocol, we used the Quality Assessment with Diverse Studies (QuADS) critical appraisal tool instead, as it was better aligned with the design and reporting characteristics of the included articles.

### Information Source and Search Strategy

We developed a search strategy and sought full-text articles published in English between January 2019 and December 2023 across three databases: PubMed, Web of Science, and Scopus. Our search strategy incorporated three terms: wearable device, remote monitoring, and chronic disease, as well as relevant synonyms. We used the AND operator between the keywords and the OR operator within the synonyms (Multimedia Appendix 1).

### Eligibility Criteria

To ensure a concentrated analysis of wearable devices for remote monitoring of chronic diseases, we established the following eligibility criteria.

- Inclusion criteria:
  - Articles that used wearable devices for the remote monitoring of chronic diseases
  - Articles that used sensors for the remote collection of health parameters
  - Original research articles published in peer-reviewed journals
- Exclusion criteria:
  - Articles that used unobtrusive, implantable, or nonwearable devices or sensor types
  - Articles that did not involve remote monitoring or data collection
  - Nonjournal publications
  - Review articles

### Data Management and Extraction

We conducted data extraction through regular team communication while reviewing the articles’ titles, abstracts, and full texts. We engaged in thorough interranker discussions to resolve discrepant findings. For data extraction, we used a well-organized spreadsheet (Microsoft Excel) with the following parameters: author names, year of publication, country, study design, study phase, device name, device status, device type, sensor types, health parameters, body locations, and medical applications (Multimedia Appendix 2).

### Standardized Terminology

#### Overview

**Figure 1.**
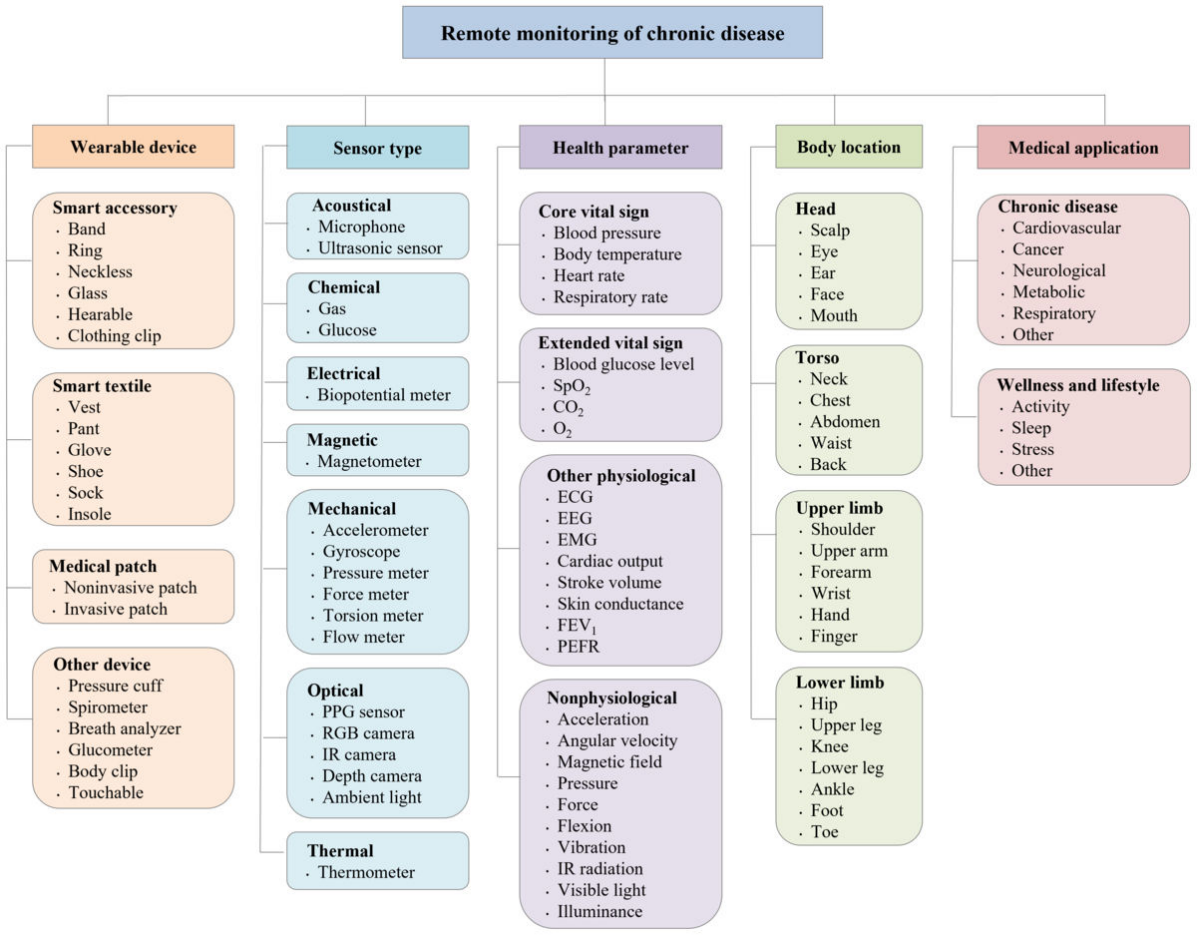
Our standardized terminology framework outlines 5 categories used to guide the systematic analysis of articles on remote monitoring of chronic diseases. CO_2_: carbon dioxide; ECG: electrocardiography; EEG: electroencephalography; EMG: electromyography; FEV_1_: forced expiratory volume in the first second; IR: infrared; PEFR: peak expiratory flow rate; PPG: photoplethysmographic; O_2_: oxygen; RGB: red, green, blue; SpO_2_: peripheral oxygen saturation.

We developed a standardized terminology based on existing evidence supplemented by insights from our research team. Our terminology encompasses five predefined categories: wearable device, sensor type, health parameter, body location, and medical application (Figure 1). We systematically organized the articles by these categories to facilitate a thorough analysis of the available evidence. The following sections detail the rationale and evidence.

#### Wearable Device

We defined a wearable device as an Internet of Things–enabled electronic device that users wear externally on the body to monitor and collect health parameters, either continuously or sporadically, and to transmit data remotely for health monitoring purposes [43]. Our definition encompasses noninvasive and minimally invasive devices that users wear externally and that do not require surgical implantation [44]. We explicitly excluded fully implantable devices, environmental sensors, and purely handheld devices not designed for body attachment [45]. We categorized wearable devices into four types:

- Smart accessory: a stand-alone electronic device that users wear continuously on the body, including bands (smartwatches, wristbands, armbands, and straps), rings, necklaces, glasses, hearables, and clothing clips
- Smart textile: a device that integrates into everyday clothing items such as vests, pants, gloves, shoes, socks, and insoles
- Medical patch: a device with adhesive skin contact or a minimally invasive needle
- Other device: a device that users wear sporadically for specific measurements, such as pressure cuffs, spirometers, breath analyzers, glucometers, body clips, and touchable devices, which can remotely transmit data

#### Sensor Type

Sensor types refer to the categories of sensing technologies that wearable devices use for remote monitoring of chronic diseases, grouped by the physical or chemical properties they measure (ie, acoustical, chemical, electrical, magnetic, mechanical, optical, and thermal) [46].

#### Health Parameter

Health parameters are measurable indicators that provide insights into an individual’s health status, including core vital signs, extended vital signs, other physiological data, and nonphysiological data [14].

#### Body Location

Body location specifies the outer body regions for sensor placement to collect health parameters. On the basis of the classification proposed by Kim et al [47], we grouped these regions into four categories: head, torso, upper limb, and lower limb.

#### Medical Application

Medical applications refer to the specific uses of wearable devices and sensors designed to monitor, manage, or support health conditions. The primary application of wearable devices focuses on chronic disease management while optionally offering wellness and lifestyle tracking, which can supplement disease monitoring. After identifying more than 10 distinct diseases, we categorized them into five groups based on their physiological systems: cardiovascular (eg, heart diseases and hypertension), cancer, neurological (eg, stroke, epilepsy, neurodegenerative disorders, and peripheral neuropathy), metabolic (eg, diabetes and obesity), respiratory (eg, asthma, chronic obstructive pulmonary disease), and other diseases [48,49]. This allows us to focus on broader applications of wearable devices across various diseases rather than focusing solely on specific conditions.

### Quality Appraisal

Our review encompassed various study designs, including user-centered design, observational, experimental, and mixed methods articles. To assess the quality of these diverse articles, we used the QuADS critical appraisal tool [50]. The QuADS includes 13 criteria rated on a scale ranging from 0 to 3 (0=not at all, 1=very slightly, 2=moderately, and 3=complete), with a total quality score ranging from 0 to 39 (Multimedia Appendix 3) [51].

## Results

### Identified Articles

**Figure 2.**
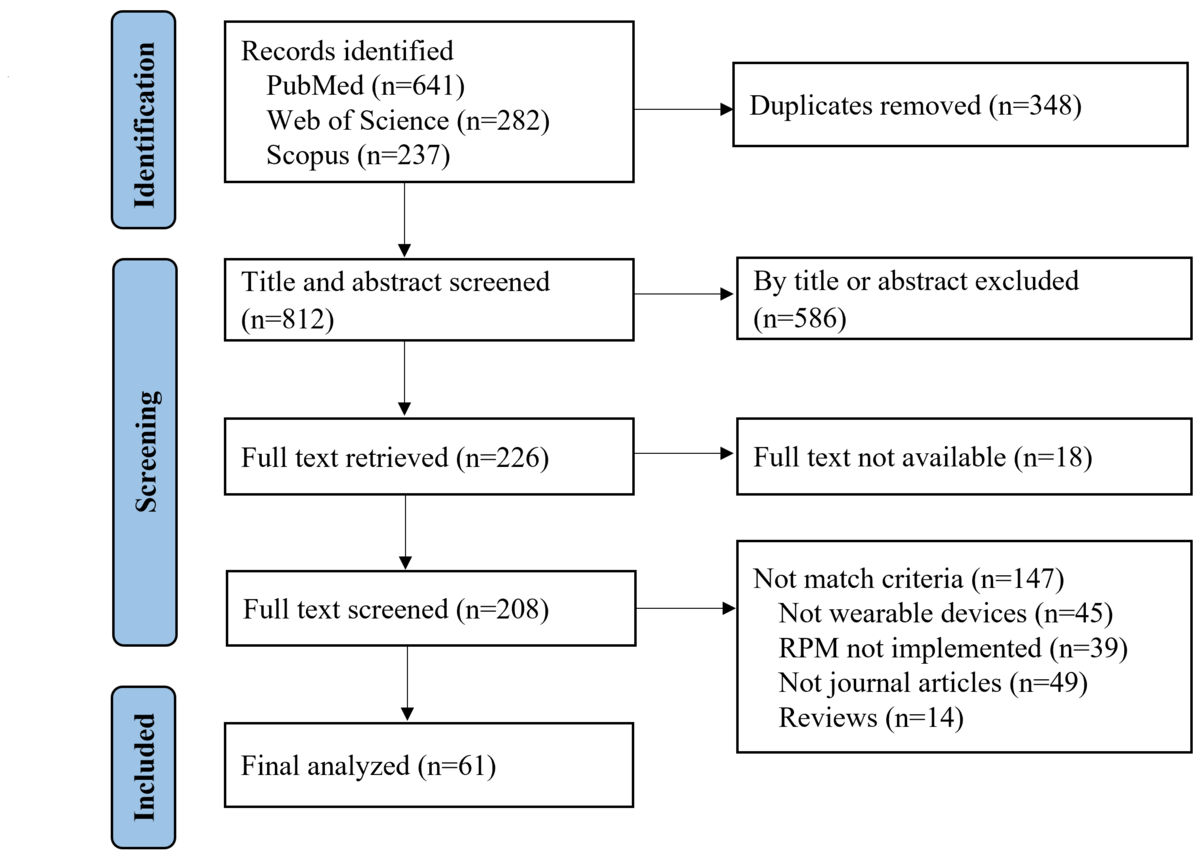
PRISMA (Preferred Reporting Items for Systematic Reviews and Meta-Analyses) flowchart describing the article selection process for this review. RPM: remote patient monitoring.

We identified 1160 articles from the 3 electronic databases. After removing duplicates, we considered 812 articles for title and abstract screening and excluded 586. Of the 226 articles screened, 18 were unavailable in full text. We thoroughly assessed 208 full-text articles for eligibility and excluded 147 that did not meet the inclusion and exclusion criteria. Finally, we included 61 articles (Figure 2).

### Characteristics of Included Articles

The included articles span Europe, North America, and the Asia-Pacific region. A total of 29 (48%) articles were conducted in Europe, with Italy contributing the most [25,31,52-55], followed by the United Kingdom [54,56-59]. North America ranks second, with nearly all articles conducted in the United States [26-28,32,34,60-73], except one article conducted in Canada [74].

Researchers used various research methodologies and study designs. Observational designs were the most prevalent (29/61, 48%), followed by 13 (21%) articles using user-centered design paired with experimental validation. Nine (15%) articles relied solely on experimental methods, and 7 (11%) articles focused exclusively on user-centered design. Only 3 (5%) articles adopted a mixed methods approach. In line with these designs, most articles (52/61, 85%) aimed to develop RPM systems and conduct feasibility and usability testing. In contrast, only 9 (15%) articles focused on late-stage validation and clinical efficacy evaluation.

We identified 74 instances of wearable devices used across the included articles. Of these, 28 (38%) instances involved dedicated medical devices approved by regulatory bodies, such as the US Food and Drug Administration (FDA) or the European Conformity Medical Device Regulation (CE-MDR). Most of these (24/28, 86%) collected vital signs and other physiological parameters. Twenty-six (35%) instances were consumer-grade wellness devices lacking medical certification, nearly all (23/26, 88%) designed as bands. Fourteen (19%) instances represented the early-stage development of research prototype devices. Additionally, 6 (8%) used consumer-grade devices with certified medical functions (FDA/CE-MDR/HIPAA [Health Insurance Portability and Accountability Act] approved) [29,34,65,70,75,76] (Table 1).

**Table 1.** Summary of the characteristics of the included articles (N=61).

Characteristics and category	Articles, n (%)^a^	Reference
Location		
Europe	29 (48)	[24,25,29,31,33,35,52-59,75-89]
North America	20 (33)	[26-28,32,34,60-74]
Asia-Pacific	12 (20)	[90-101]
Study design^b^		
User-centered design	7 (11)	[27,55,71,77,85,96,97]
Observational	29 (48)	[24,28,29,32,33,35,53,57,58,61-64,67,69,70,72-74,76,79,81-83,86,90,94,95,99]
Experimental	9 (15)	[34,54,66,68,75,78,89,93,100]
User-centered design with experimental validation	13 (21)	[25,31,52,56,59,60,65,84,87,88,91,92,98]
Mixed methods	3 (5)	[26,80,101]
Study phase^c^		
Technology development and piloting	21 (34)	[26,27,31,52,55,56,59-61,65,71,77,84,85,87,88,91,92,96-98]
Feasibility and usability testing	31 (51)	[24,25,28,32,33,35,54,57,58,63,64,66-70,72-74,76,78-82,90,93,95,99-101]
Clinical validation	5 (8)	[29,62,75,86,94]
Clinical outcome evaluation	4 (7)	[34,53,83,89]
Device status^d^		
Dedicated medical device (FDA^e^/CE-MDR^f^ approved)	28 (38)	[28,32,53,55,57,58,60-62,64,66,68,71,73,79-83,86,88,93,100]
Consumer-grade device with certified medical function (FDA/CE-MDR/HIPAA^g^ approved)	6 (8)	[29,34,65,70,75,76]
Consumer-grade wellness device	26 (35)	[24,26,29,35,54,57,61,63-65,67,69,72,78,80,84,87,89-91,95,96,99]
Research prototype device	14 (19)	[24,25,27,52,54,56,58,59,77,80,85,92,98,101]

a Percentages may not total 100% due to rounding.

b The study design describes the methodological approach used in each article.

c The study phase indicates the stage of development or evaluation addressed.

d The regulatory status reflects the device’s classification at the time of the study, not its current status. Some articles used multiple devices; therefore, device status is reported based on 74 wearable device instances.

e FDA: US Food and Drug Administration.

f CE-MDR: the European Conformity Medical Device Regulation.

g HIPAA: Health Insurance Portability and Accountability Act.

### Wearable Device

**Figure 3.**
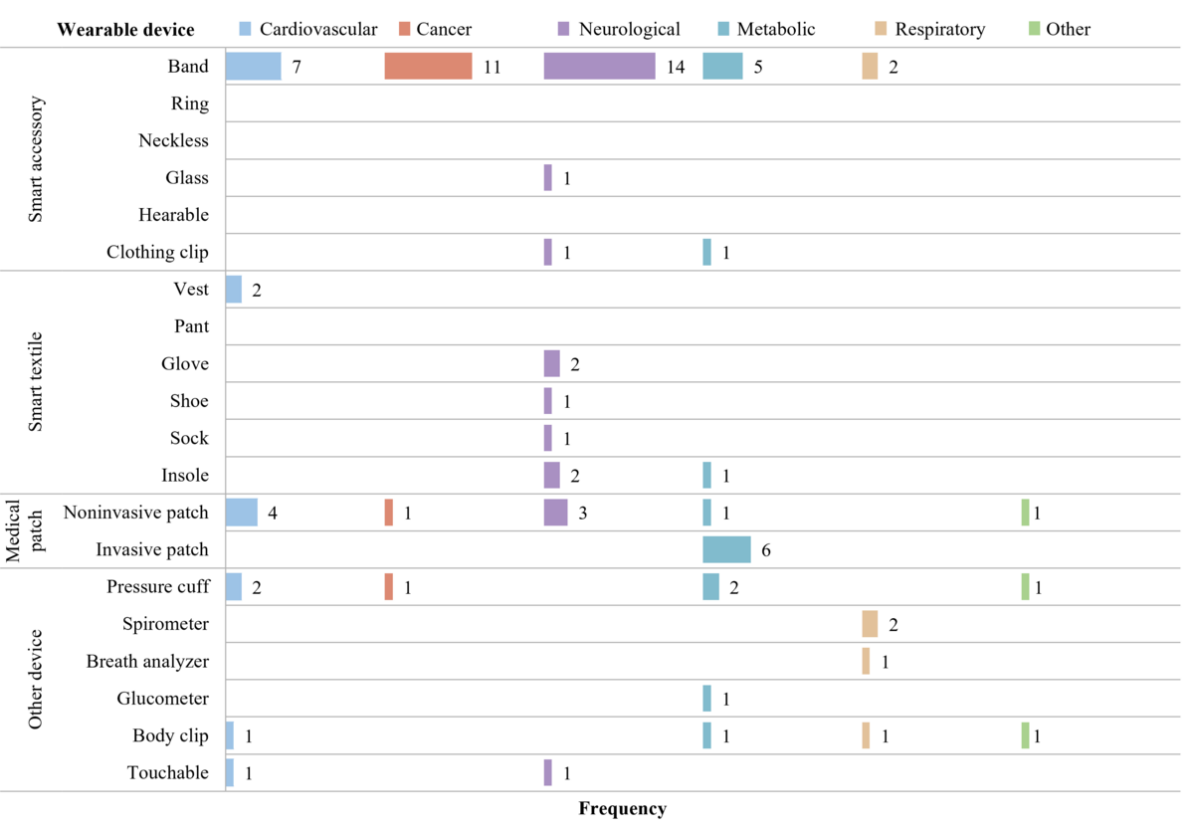
Occurrence of wearable devices by categories summarized in Figure 1. Rows represent the number of articles (N=61) that use each wearable device; colored columns (disease categories) may exceed N because some articles report on multiple devices.

We identified 16 distinct wearable devices for remote monitoring of chronic diseases (Figure 3). Bands, noninvasive patches, and pressure cuffs emerged as the most frequently used devices across various chronic diseases, appearing in 39 (64%), 10 (16%), and 6 (10%) articles, respectively. Neurological disease monitoring used 9 devices, with bands being the most common [24,25,31,35,54,56,65,72,73,87,89,91,98,101]. Researchers used 8 distinct wearable devices for metabolic diseases, with invasive patches [32,53,55,60,80,83] being exclusive to this category and the most frequently used, followed by bands [29,55,80,95,96]. Cardiovascular monitoring involved 6 wearable devices, primarily bands [67,69,75,76,84,93,94] and noninvasive patches [33,62,71,85]. For cancer monitoring, the articles almost exclusively reported on bands [34,58,63,64,78,81,82,90,97,99,100].

### Sensor Type

The wearable devices feature 14 sensor types. Accelerometers, PPG sensors, biopotential meters, pressure meters, and thermometers are most frequently used to monitor chronic conditions, appearing in 39 (64%), 18 (30%), 17 (28%), 11 (18%), and 9 (15%) articles, respectively. The wearable devices typically monitor neurological diseases using a diverse array of 10 sensors, preferring accelerometers [24,25,27,31,35,54,56,65,68,72,73,87,89,91,98,101], gyroscopes [24,25,27,31,35,54,56,65,68,73,91,98,101], and magnetometers [24,25,31,56,65,73,98] being the most common. Researchers used force meters [54,56], torsion meters [27,98], and wearable cameras [70], exclusively for neurological diseases.

**Figure 4.**
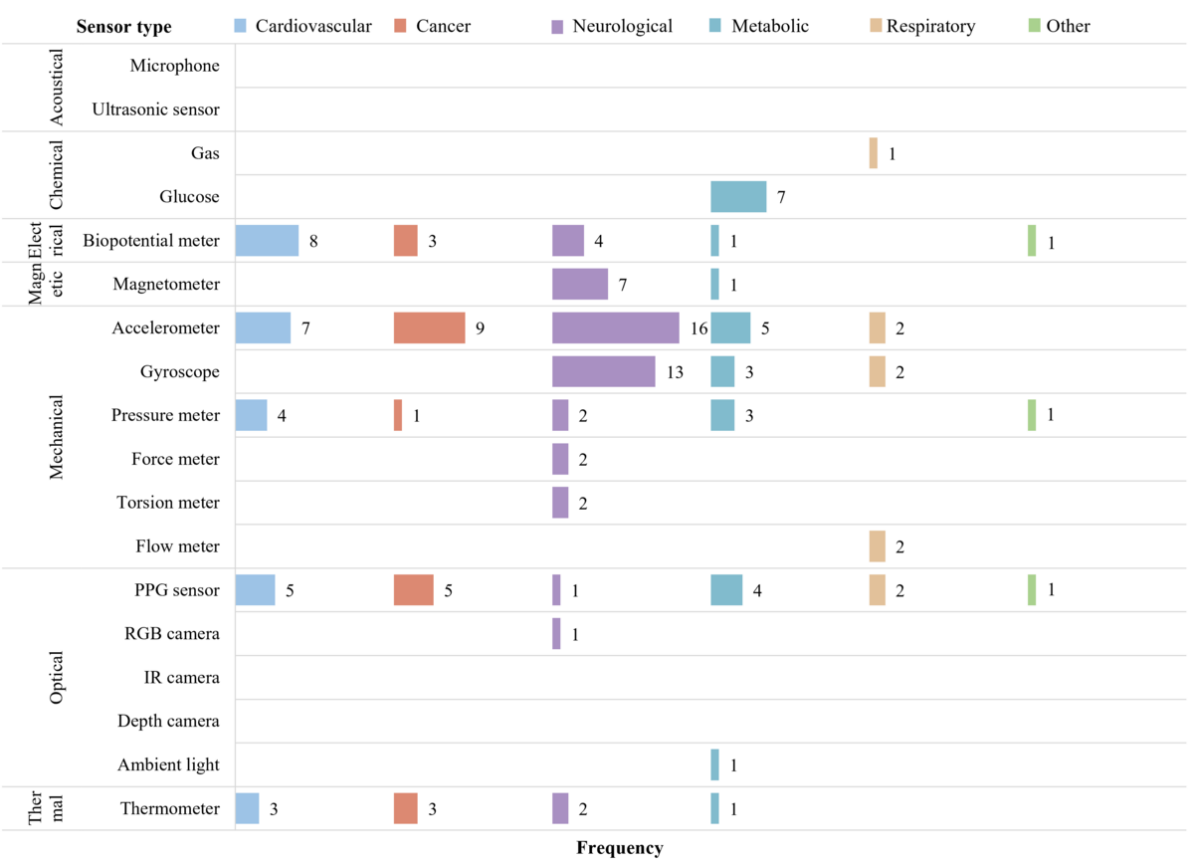
Occurrence of sensor types by categories summarized in Figure 1. Rows represent the number of articles (N=61) that use each sensor; colored columns (disease categories) may exceed N because some articles report on multiple sensors. IR: infrared; PPG: photoplethysmographic; RGB: red, green, blue.

We identified sensors such as biopotential meters [33,62,71,75,77,79,85,86], accelerometers [62,67,71,79,84,86,93], PPG sensors [67,69,74,84,93], pressure meters [74,76,77,94], and thermometers [62,71,93] as key sensors for the remote monitoring of cardiovascular diseases, ranked by their usage frequency. Similarly, we identified that these sensors monitor cancer, although their frequency varies. Accelerometers [34,58,63,64,81,82,90,99,100], PPG sensors [78,81,82,97,99], biopotential meters [64,81,82], thermometers [81,82,97], and pressure meters [64] are the most common. Additionally, our analysis showed that the articles used glucose sensors [32,53,55,60,80,83,96] exclusively and frequently for monitoring metabolic diseases (Figure 4).

### Wearable Device, Sensor Type, and Chronic Disease Mapping

**Figure 5.**
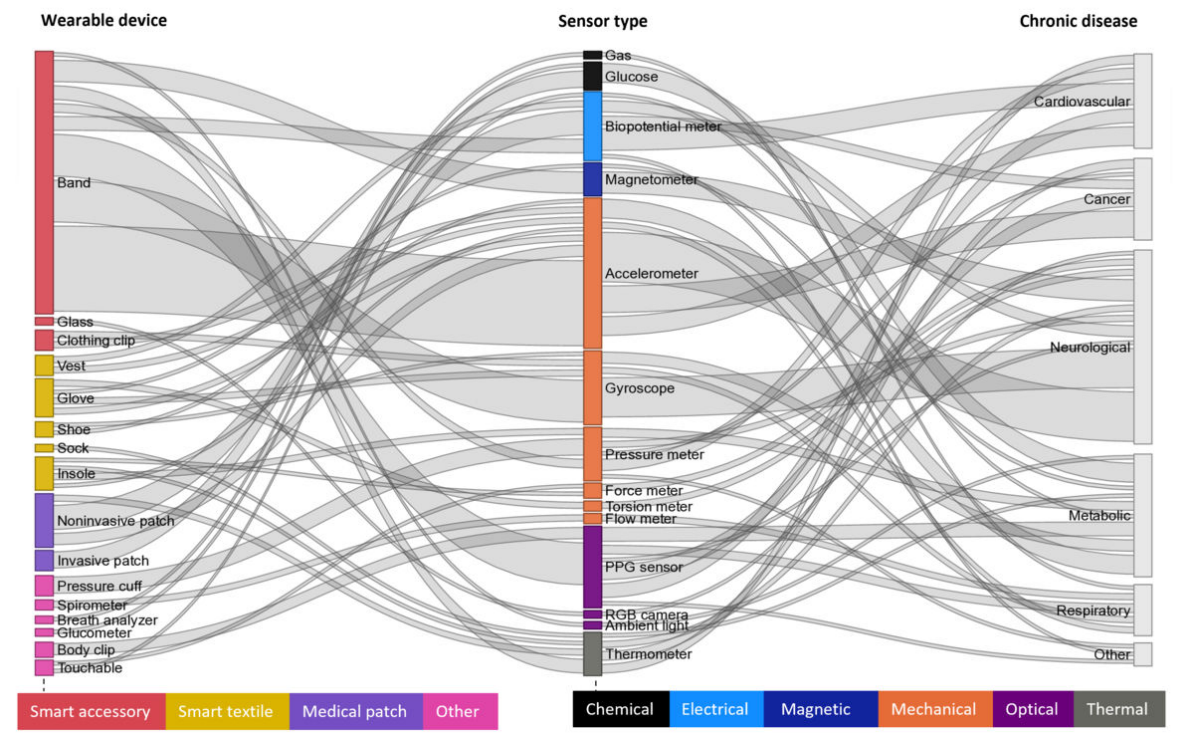
A Sankey diagram illustrating the mapping between wearable devices, sensor types, and the chronic diseases targeted for remote monitoring. Node and link widths reflect match frequency, and node colors correspond to wearable device and sensor types. PPG: photoplethysmographic; RGB: red, green, blue.

A Sankey diagram visually illustrates the relationships among wearable devices, sensor types, and the corresponding chronic diseases they monitor (Figure 5). Bands have emerged as the most widely used wearable devices, integrating 8 sensor types. They primarily incorporate accelerometers [24,25,31,35,54,56,65,72,73,87,89,91,98,101], gyroscopes [24,25,31,35,54,56,65,73,91,98,101], and magnetometers [24,25,31,56,65,73,98] to facilitate remote monitoring of neurological diseases. They also often feature PPG sensors [29,54,55,57,61,67,69,78,80-82,84,93,96,97,99] and biopotential meters [64,75] to track heart rate and ECG. Moreover, bands are increasingly equipped with other sensor types such as electrodermal activity (EDA) biopotential meters [54,81,82], skin thermometers [54,81,82,93,97], oscillometric blood pressure meters [76,94], barometric altimeters [80], and ambient light sensors [80], enabling remote monitoring of skin conductance, body temperature, blood pressure, atmospheric pressure, and illumination, respectively.

Noninvasive medical patches incorporate accelerometers [58,62,71,80] and thermometers [62,71], in addition to common types of biopotential meters such as ECG [33,62,71,80,88], electroencephalography [66,92], electromyography [26], and impedance [85]. Notably, glucose sensors are the only sensor type integrated into minimally invasive devices [32,53,55,60,80,83]. Furthermore, traditional pressure cuffs, such as the blood pressure monitors iHealth (iHealth Labs Inc) [88,96] and Withings (Withings) [29], now transmit their data remotely. Additionally, pulse oximeters such as iHealth [96] and Onyx (Nonin Medical Inc) [88] enhance the functionality of traditional body clips by enabling the remote transmission of peripheral oxygen saturation (SpO_2_) and heart rate data.

### Health Parameter

**Figure 6.**
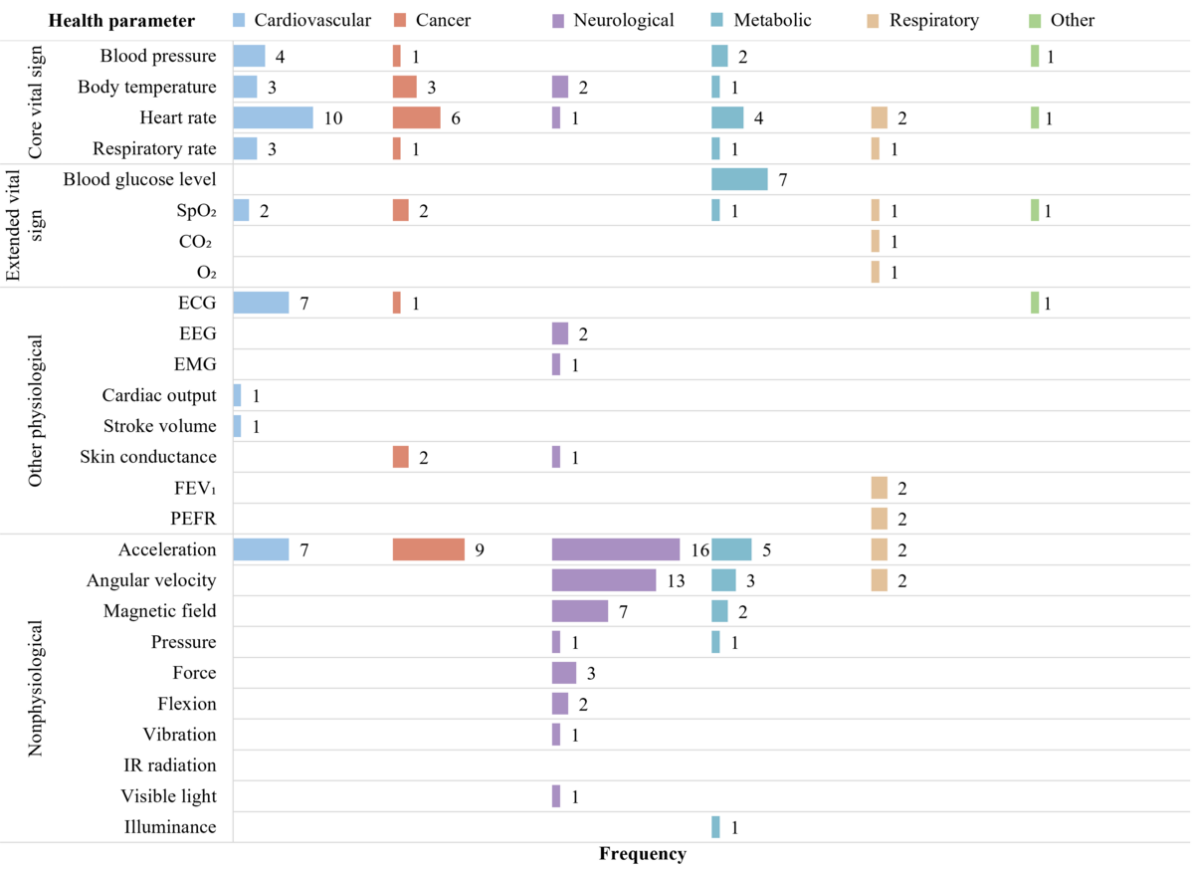
Occurrence of health parameters by categories summarized in Figure 1. Rows represent the number of articles (N=61) that collect each health parameter; colored columns (disease categories) may exceed N because some articles collect multiple health parameters. CO_2_: carbon dioxide; ECG: electrocardiography; EEG: electroencephalography; EMG: electromyography; FEV_1_: forced expiratory volume in the first second; IR: infrared; PEFR: peak expiratory flow rate; PPG: photoplethysmographic; O_2_: oxygen; RGB: red, green, blue; SpO_2_: peripheral oxygen saturation.

Sensors gather a total of 25 health parameters for remote monitoring of chronic diseases (Figure 6). Acceleration is a key health parameter for monitoring various chronic diseases, as indicated by 39 (64%) articles. Specifically, it is the most commonly collected health parameter in the remote monitoring of neurological diseases [24,25,27,31,35,54,56,65,68,72,73,87,89,91,98,101] and cancer [34,58,63,64,81,82,90,99,100].

The articles consistently reported on monitoring heart rate, body temperature, blood pressure, and SpO_2_, as reported in 24 (39%), 9 (15%), 8 (13%), and 7 (11%) articles, respectively. These core and extended vital signs are essential health parameters for remote monitoring of cardiovascular, cancer, and metabolic diseases. On the other hand, articles primarily monitor neurological diseases using nonphysiological parameters. Furthermore, metabolic monitoring focuses on core and extended vital signs and nonphysiological parameters (Figure 6).

### Body Location

**Figure 7.**
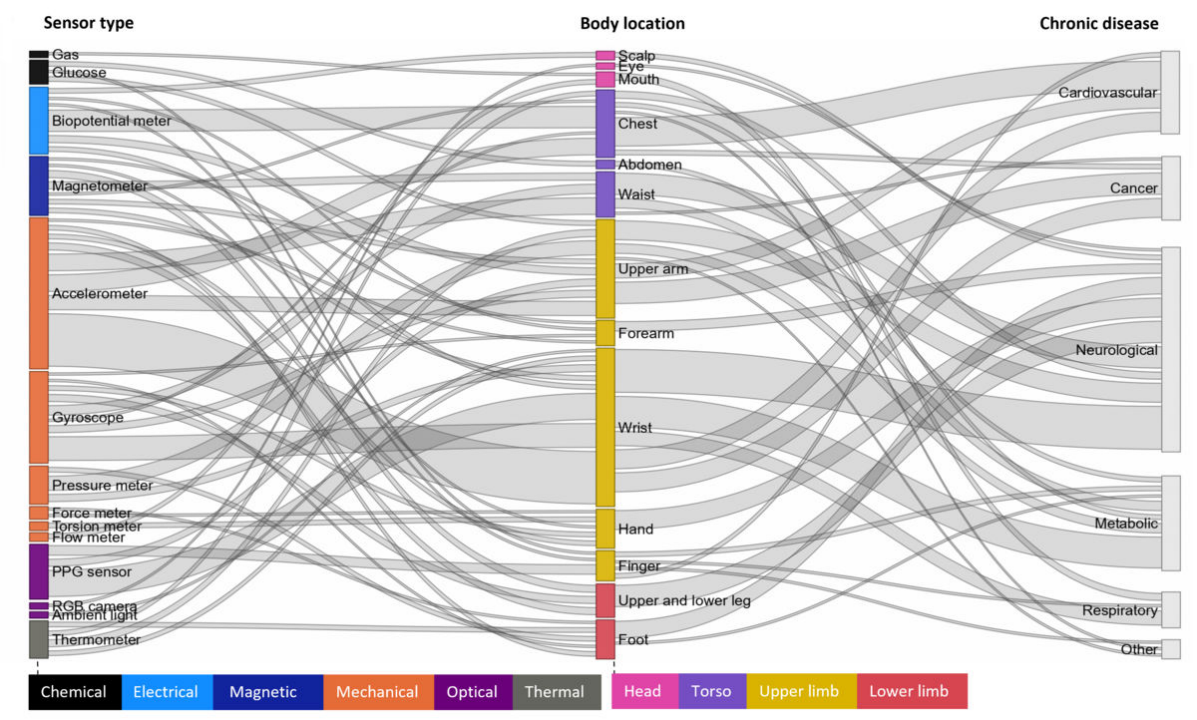
A Sankey diagram illustrating body locations for sensor placement in the remote monitoring of various chronic diseases. Node and link widths reflect match frequency, and node colors correspond to sensor type and body locations. PPG: photoplethysmographic; RGB: red, green, blue.

The wrist is the most frequent body location for sensor placement, comprising 8 different sensor types. A Sankey diagram illustrates the linkage between accelerometers, PPG sensors, and gyroscopes to the wrist, highlighting that these sensor types are mostly positioned on the wrist (Figure 7). The upper arm is the second-most frequent body location, comprising 8 sensor types, including pressure meters [29,64,74,77,88,96] and accelerometers [35,56,81,82,93,98]. The chest is the third-most common body location, accommodating 5 sensor types. Researchers commonly place biopotential meters [33,62,71,79,80,88] here to monitor cardiovascular diseases. Additionally, the waist is an ideal location for IMU sensors, including accelerometers [64,65,68,72,73,80,95], gyroscopes [65,68,73,80], and magnetometers [65,73,95] for monitoring neurological, metabolic, and cancer diseases. Furthermore, researchers place various sensors on the hand, forearm, upper, and lower legs to monitor neurological diseases [26,27,31,56,73,98].

### Medical Application

**Figure 8.**
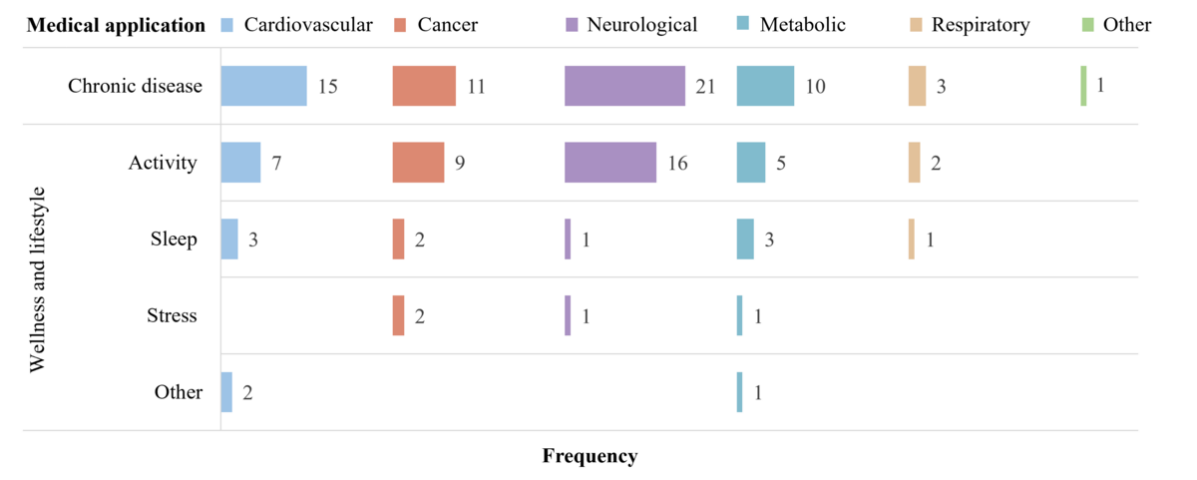
Frequency of key medical applications by categories summarized in Figure 1. Rows represent the number of articles (N=61 for chronic disease monitoring and N=39 for wellness and lifestyle applications); colored columns may exceed N because some articles report on multiple wellness or lifestyle applications.

The articles primarily highlighted the use of wearable devices for monitoring specific chronic diseases as their primary medical application, with the option to include wellness and lifestyle monitoring. Among chronic diseases, neurological diseases are the primary focus, accounting for more than one-third (21/61, 34%) of the articles. Cardiovascular diseases follow, with 15 (25%) articles, while wearable devices monitored cancer, metabolic diseases, and respiratory diseases in 11 (18%), 10 (16%), and 3 (5%) articles, respectively (Figure 8).

Furthermore, more than half (39/61, 64%) of the articles targeted wellness and lifestyle applications alongside disease-specific metrics. Activity tracking is a fundamental wellness and lifestyle metric collected across 39 (100%) articles. Notably, activity remains essential for monitoring neurological diseases. Furthermore, sleep monitoring is another application widely reported in 10 (26%) articles for various chronic conditions. For cancer, neurological, and metabolic diseases, the articles described remote stress-level monitoring (Figure 8).

### Quality Assessment of Included Articles

More than half of the included articles fully met 11 of the 13 QuADS criteria, receiving the top rating (Complete) on the QuADS scale (Table 2). Specifically, 56 (92%) and 54 (89%) articles clearly described the data collection procedure and stated the research aim, respectively. Fifty-two (85%) articles provided detailed descriptions of the research setting and population, used an appropriate format and content for the data collection tool, and applied proper methods of analysis to address the research aim. In contrast, only 15 (25%) articles thoroughly addressed stakeholder involvement in the design and conduct of the article, while 11 (18%) articles did not report stakeholder involvement at all (Multimedia Appendix 3).

**Table 2.** Quality assessment scores for the 13 Quality Assessment with Diverse Studies (QuADS) criteria [50].

QuADS criteria	Rating scale^a^, n (%)^b^			
	Not at all	Very slightly	Moderately	Complete
Theoretical or conceptual underpinning to the research	0 (0)	1 (2)	25 (41)	35 (57)
Statement of research aim/s	0 (0)	1 (2)	6 (10)	54 (89)
Clear description of research setting and target population	0 (0)	2 (3)	7 (11)	52 (85)
Study design appropriate to address the stated research aim/s	0 (0)	0 (0)	17 (28)	44 (72)
Appropriate sampling to address the research aim/s	2 (3)	5 (8)	31 (51)	23 (38)
Rationale for choice of data collection tool/s	3 (5)	5 (8)	16 (26)	37 (61)
Format and content of data collection tool appropriate to research aim/s	0 (0)	0 (0)	9 (15)	52 (85)
Description of data collection procedure	0 (0)	0 (0)	5 (8)	56 (92)
Recruitment data provided	4 (7)	5 (8)	14 (23)	38 (62)
Justification for analytic method selected	7 (11)	5 (8)	18 (30)	31 (51)
Method of analysis appropriate to answer the research aim/s	2 (3)	0 (0)	7 (11)	52 (85)
Evidence that stakeholders were considered in design/conduct	11 (18)	12 (20)	23 (38)	15 (25)
Strengths and limitations critically discussed	5 (8)	0 (0)	23 (38)	33 (54)

a Each cell indicates the number of articles (N=61) that met the corresponding QuADS criterion at the given rating scale.

b Percentages may not total 100% due to rounding.

## Discussion

### Principal Findings and Interpretation

We identified the wearable devices, sensor types, health parameters, body locations, and medical applications for remote monitoring of various chronic diseases. We found a clear trend toward wrist-worn bands primarily tracking acceleration, followed by core and extended vital signs. These devices mainly monitor neurological and cardiovascular diseases.

Wearable devices predominantly come in the form of bands, a trend driven by consumer electronics giants such as Fitbit [34,55,67,72,90], Apple [55,69,84,99], Garmin [57,80], Huawei [94,96], Samsung [99], Withings [75], Omron HeartGuide [76], and Misfit [63]. These brands offer a diverse range of wearable devices, including smartwatches, wristbands, armbands, and straps. However, most consumer-grade wellness devices lack medical certification, and 23 of 26 are bands. This finding urges future researchers and clinicians to look beyond wearability and user-friendliness and to consider regulatory status. The emergence of consumer-grade wellness devices with certified medical functions (FDA/CE-MDR approval), although still a minority, represents a significant development for RPM systems by addressing the need for clinical validation.

A wide range of wearable devices integrated accelerometers to track acceleration, which emerged as the most frequently monitored and clinically relevant health parameter across multiple chronic diseases. This is because acceleration offers valuable insights for designing activity plans that support wellness and slow disease progression [102]. Our findings further highlight its particular prevalence in the remote monitoring of neurological disorders and cancer. This aligns with previous research, demonstrating that tracking acceleration enables researchers to gain a deeper understanding of movement patterns, serving as key indicators of Parkinson disease [103-105], and to assess motor function and recovery following a stroke [106,107]. Acceleration is also a key health parameter for monitoring physical fitness levels and evaluating treatment effectiveness in patients with cancer [108].

Vital sign monitoring remains a cornerstone for the remote management of chronic diseases [109], as it provides critical insights into a patient’s overall physiological status [110] and serves as an early warning signal for detecting and preventing patient deterioration [111]. Our findings support this principle in the context of wearable devices, revealing that articles frequently use PPG sensors, biopotential meters, pressure meters, and thermometers to track core and extended vital signs, including heart rate, body temperature, blood pressure, and SpO_2_.

The wrist is the dominant body location for sensor placement, primarily due to ongoing technological innovations in bands, which improve comfort and usability [112] while enabling the integration of key sensor types, such as accelerometers and PPG sensors [113,114]. When data quality is essential, researchers often choose the upper arm to minimize motion artifacts that corrupt health parameters, compared to the more dynamic wrist and hand [115]. Sensor placement is specific to the health parameter. The chest remains the major site for placing biopotential meters (ECG tracking), which reflects clinical practice [116]. Conversely, PPG sensors do not perform well when placed on the chest, likely due to factors such as skin type and hair density [117-120]. To quantify motor symptoms in the limbs, researchers monitoring neurological diseases often place IMUs on the extremities (eg, the upper arm, forearm, hand, and leg) [121-123].

Wearable devices primarily target neurological and cardiovascular diseases. A recent report from the Institute for Health Metrics and Evaluation supports our findings, identifying neurological diseases as the leading global cause of disease burden and disability, affecting 3.4 billion people, and surpassing the impact of cardiovascular diseases [124,125]. The effectiveness of wearable-based RPM systems for early prediction and prevention is mainly due to their ability to capture quantifiable biomarkers, such as gait analysis, tremors, speech patterns, and cognitive functions. Alongside this, researchers continue to use wearable devices to monitor cardiovascular diseases, which are the leading cause of death globally [126].

Beyond chronic disease monitoring, there is a significant emphasis on wellness and lifestyle monitoring. Approximately two-thirds of the articles track physical activities, such as counting steps and determining movement. Additionally, movement-related parameters, such as tremors, motor symptom assessment, and muscle vibration, are essential for diagnosing neurological diseases. This aligns with the WHO report, which states that promoting healthy behaviors or responding to warning signs can prevent 80% of chronic diseases [127]. Furthermore, our results align with previous research, demonstrating that regular physical activity is essential for managing chronic diseases [128,129], preventing the onset of new diseases [130-132], reducing medication needs [133,134], and enhancing quality of life [135-138]. This emphasizes the importance of setting physical activity plans as a strategy to prevent the progression of chronic diseases.

Another application of this lifestyle approach is the growing focus on remote sleep monitoring. Evidence from the Centers for Disease Control and Prevention [139] and the Population Reference Bureau [140] supports our findings, highlighting the strong association between poor sleep and various chronic diseases, including cardiovascular and metabolic disorders. As a result, we emphasize the importance of monitoring and addressing sleep problems to prevent deterioration in health and promote healthy behaviors in patients with chronic conditions.

### Comparison With Prior Work

Several systematic reviews examined the use of wearable devices for remote monitoring of chronic diseases. Some reviews evaluated the impact of wearable-based RPM systems on patient outcomes [36,41,141]. However, these articles do not specify the wearable devices, sensor types, body locations, or health parameters. Other reviews analyze medical applications of RPM systems [142,143], but they do not provide cross-disease mapping of wearable devices, sensor types, body locations, or monitored health parameters. Some reviews focus on specific applications, such as primary health care [37], rehabilitation [14], or physical activity monitoring [144], but do not compare wearable devices or parameters across health conditions.

In contrast, our systematic review provides a framework for wearable devices, sensor types, body locations, health parameters, and medical applications. We visualize relationships among wearable devices, sensor types, and chronic diseases, as well as between sensor types, body locations, and chronic diseases. Building on the recommendation from Cajamarca et al [145], we argue that managing multiple chronic conditions represents a forward-looking approach to address the global rise in multimorbidity. For instance, data from Watson et al [146] show that 51.4% of US adults live with multiple chronic conditions. By identifying shared components across chronic conditions, our findings provide foundational evidence for designing generalized wearable-based RPM systems. Such multifunctional systems cost-effectively monitor multiple chronic conditions simultaneously, moving beyond disease-specific solutions [147].

Martins et al [148] highlighted the need for common standards to address ongoing interoperability challenges across diverse RPM systems. Identifying shared components across pilot projects, commercial products, and CE/FDA-approved wearable devices represents a critical first step toward the semantic harmonization of clinical data across chronic conditions [149]. Our work fosters a deeper understanding of the increasing diversity of RPM systems, supports the assessment of Fast Healthcare Interoperability Resources requirements, encourages performing concept mapping, and promotes the development of extensions to medical terminologies, including International Classification of Diseases, 11th Revision [150], Systematized Nomenclature of Medicine—Clinical Terms [151], and Logical Observation Identifiers Names and Codes [152].

### Limitations and Future Research Directions

This review has several limitations. First, we limited the scope to wearable devices used for the remote monitoring of chronic diseases, excluding unobtrusive or implantable sensors, as well as articles that do not implement RPM systems. While this allows for a more targeted analysis, it may reduce the generalizability of the results to broader sensor technologies. Second, most of the included articles (52/61, 85%) focused on developing RPM systems and conducting early-stage feasibility and usability assessments. Although the field demonstrates strong innovation, it lacks substantial late-stage validation and evaluation of clinical impact on patient outcomes. Consequently, our findings are primarily based on reported device usage frequencies in early-stage and feasibility studies, rather than on demonstrated clinical outcomes. Therefore, readers should not interpret the frequency of a device’s appearance in this review as evidence of its clinical effectiveness. Third, we did not evaluate technical aspects, such as long-term device reliability, low power consumption, or the impact of motion artifacts on sensor accuracy. While these factors significantly affect the long-term monitoring of chronic diseases, they fall outside the predefined objectives of this review and require further investigation in future research.

Given this predominance of early-stage feasibility articles, we strongly recommend a shift in research priorities. Future work should prioritize late-stage clinical trials that use medically certified wearable devices. Such articles are essential for rigorously evaluating the clinical efficacy, data privacy, and system maturity, and the impact of motion artifacts, thereby moving RPM systems beyond proof-of-concept toward evidence-based implementation.

### Conclusions

This review confirms that the most frequent devices for remote monitoring of chronic diseases are bands, with the wrist being the preferred body location, followed by the upper arm and the chest. The most common sensor types are accelerometers, PPG sensors, and biopotential meters, which primarily collect health parameters such as acceleration, heart rate, and body temperature. Key applications of wearable devices include monitoring neurological and cardiovascular diseases, tracking activity, and assessing sleep quality. Overall, these findings highlight the need for a foundational road map for designing generalized RPM systems that can manage multimorbidity and support standardized terminology to enhance interoperability across digital health systems.

## Supplementary material

10.2196/74071 Multimedia Appendix 1 Search strategy used for identifying articles on wearable devices for remote monitoring of chronic diseases.

10.2196/74071 Multimedia Appendix 2 Complete dataset extracted from the included articles.

10.2196/74071 Multimedia Appendix 3 Quality appraisal of included articles using the 13 Quality Assessment With Diverse Studies criteria.

10.2196/74071 Checklist 1 PRISMA checklist.

## Abbreviations

CE-MDR: European Conformity Medical Device Regulation
ECG: electrocardiography
FDA: US Food and Drug Administration
HIPAA: Health Insurance Portability and Accountability Act
IMU: inertial measurement unit
PPG: photoplethysmographic
PRISMA: Preferred Reporting Items for Systematic Reviews and Meta-Analyses
QuADS: Quality Assessment With Diverse Studies
RPM: remote patient monitoring
SpO_2_: peripheral oxygen saturation
WHO: World Health Organization
