# Supplementary material for: Wearable Devices for Remote Monitoring of Chronic Diseases: Systematic Review
Source: JMIR Mhealth Uhealth. 2026 Feb 11;14:e74071. doi: 10.2196/74071 (PMC12893647; doi:10.2196/74071)
Supplement: Multimedia Appendix 1 [file mhealth-v14-e74071-s001.docx]

**Multimedia Appendix 1. Search strategy used for identifying articles on wearable devices for remote monitoring of chronic diseases**

| **Databases** | **Search strategy** | **Search results** |
| --- | --- | --- |
| **PubMed** | ((((("Wearable Electronic Devices"*[Mesh]* **OR** "Smart wearable*"*[Title/Abstract*] **OR** "Wearable technolog*" *[Title/Abstract]* **OR** "Wearable device" *[Title/Abstract]* **OR** "Wearable sensor*"*[Title/Abstract]* **OR** wearable**[Title/Abstract]* **OR** Sensor**[Title/Abstract])*  **AND**  ("Telemedicine"*[Mesh]* **OR** Telemedicine*[Title/Abstract]* **OR** eHealth*[Title/Abstract]* **OR** "Remote Monitoring" *[Title/Abstract]* **OR** "Remote continuous monitoring" *[Title/Abstract]* **OR** Telemonitoring*[Title/Abstract]* **OR** Telecare*[Title/Abstract]* **OR** Telehealth*[Title/Abstract]* **OR** mHealth*[Title/Abstract]* **OR** " Home health monitoring "*[Title/Abstract]*))  **AND**  ("Chronic Disease"*[Mesh]* **OR** "Chronic Disease"*[Title/Abstract]* **OR** "Chronic Obstructive Pulmonary Disease"*[Title/Abstract]* **OR** Diabetes*[Title/Abstract]* **OR** "Coronary heart diseases "*[Title/Abstract]* **OR** "Heart Failure"*[Title/Abstract]* **OR** Hypertension*[Title/Abstract]* **OR** "Parkinson's disease"*[Title/Abstract]* **OR** Stroke*[Title/Abstract]* **OR** Obesity*[Title/Abstract]* **OR** Cancer*[Title/Abstract]* **OR** Asthma*[Title/Abstract]* **OR** Epilepsy*[Title/Abstract]* **OR** Arthritis*[Title/Abstract]* **OR** "Alzheimer’s disease and dementia"*[Title/Abstract]* **OR** "Chronic kidney diseases"*[Title/Abstract]* **OR** Osteoporosis*[Title/Abstract]* **OR** Depression*[Title/Abstract]* **OR** "Chronic Liver Disease"*[Title/Abstract]*))  **NOT** (Review*[Publication Type]* OR "Systematic review"*[Publication Type]* OR "Meta-Analysis"*[Publication Type]*))  **AND** (English*[Language]*))  **AND** (("2019"*[Date - Publication]* : "2023"*[Date - Publication]*)) | 641 |
| **Scopus** | *TITLE-ABS-KEY* (("Wearable Electronic Devices" **OR** "Smart wearable*" **OR** "Wearable technolog*" **OR** "Wearable device" **OR** "Wearable sensor*" **OR** "wearable*" **OR** "Sensor*")  **AND**  ("Telemedicine" **OR** "eHealth" **OR** "Remote Monitoring" **OR** "Remote continuous monitoring" **OR** "Telemonitoring" **OR** "Telecare" **OR** "Telehealth" **OR** "mHealth" **OR** "Home health monitoring ")  **AND**  ( "Chronic Disease" **OR** "Chronic Obstructive Pulmonary Disease" **OR** "Diabetes" **OR** "Coronary heart diseases " **OR** "Heart Failure" **OR** "Hypertension" **OR** " Parkinson diseases" **OR** "Stroke" **OR** "Obesity" **OR** "Cancer" **OR** "Asthma" **OR** "Epilepsy" **OR** "Arthritis" **OR** "Alzheimer disease and dementia " **OR** "Chronic kidney diseases" **OR** Osteoporosis **OR** Depression **OR** "Chronic Liver Disease" ) )  **AND NOT** ("Systematic review" **OR** "Review" **OR** "Meta-Analysis")  **AND** ( *LIMIT-TO* ( PUBSTAGE,"final" ) )  **AND** ( *LIMIT-TO* ( PUBYEAR,2019) OR LIMIT-TO ( PUBYEAR,2020) OR LIMIT-TO ( PUBYEAR,2021) OR LIMIT-TO ( PUBYEAR,2022) OR LIMIT-TO ( PUBYEAR,2023) )  **AND** ( *LIMIT-TO* ( LANGUAGE,"English" ) ) | 237 |
| **Web of Sciences** | ((((*ALL=(* (Wearable Electronic Devices) **OR** (Smart wearable*) **OR** (Wearable technolog*) **OR** (Wearable device) **OR** (Wearable sensor*) **OR** (Wearable*) OR (Sensor*)))  **AND**  *ALL=*((Telemedicine) **OR** (eHealth) **OR** (Remote Monitoring) **OR** (Remote continuous monitoring) **OR** (Telemonitoring) **OR** (Telecare) **OR** (Telehealth) **OR** (mHealth) **OR** (Home health monitoring)))  **AND**  *ALL=*((Chronic Disease) **OR** (Chronic Obstructive Pulmonary Disease) **OR** (Diabetes) **OR** (Coronary heart diseases) **OR** (Heart Failure) **OR** (Hypertension) **OR** (Parkinson's disease) **OR** (Stroke) **OR** (Obesity) **OR** (Cancer) **OR** (Asthma) **OR** (Epilepsy) **OR** (Arthritis) **OR** (Alzheimer disease and dementia) **OR** (Chronic kidney diseases) **OR** (Osteoporosis) **OR** (Depression) **OR** (Chronic Liver Disease)))  **NOT** *DT=*(Review OR Editorial Material OR Meeting Abstract OR Book OR Letter))  **AND** *PY=*(2019 OR 2020 OR 2021 OR 2022 OR 2023) | 282 |
