# Supplementary material for: Wearable Devices for Remote Monitoring of Chronic Diseases: Systematic Review
Source: JMIR Mhealth Uhealth. 2026 Feb 11;14:e74071. doi: 10.2196/74071 (PMC12893647; doi:10.2196/74071)
Supplement: Multimedia Appendix 3 [file mhealth-v14-e74071-s003.docx]

**Multimedia Appendix 3: Quality appraisal of included articles using the 13 QuADS criteria.**

| **Reference, Year** | **C1** | **C2** | **C3** | **C4** | **C5** | **C6** | **C7** | **C8** | **C9** | **C10** | **C11** | **C12** | **C13** | **Total score** |
| --- | --- | --- | --- | --- | --- | --- | --- | --- | --- | --- | --- | --- | --- | --- |
| Chi [71], 2021 | 2 | 3 | 3 | 3 | 3 | 3 | 3 | 3 | 2 | 3 | 3 | 3 | 3 | 37 |
| Stehlik [62],2020 | 2 | 3 | 3 | 3 | 3 | 3 | 3 | 3 | 3 | 3 | 3 | 2 | 3 | 37 |
| Sohn [67], 2020 | 3 | 3 | 3 | 3 | 3 | 3 | 3 | 3 | 3 | 3 | 3 | 2 | 3 | 38 |
| Bleda [77], 2019 | 3 | 3 | 3 | 3 | 2 | 3 | 2 | 3 | 2 | 2 | 3 | 2 | 2 | 33 |
| Blockhaus [79], 2021 | 2 | 3 | 3 | 3 | 3 | 3 | 3 | 3 | 3 | 3 | 3 | 1 | 3 | 36 |
| Wong [93],2022 | 3 | 3 | 3 | 3 | 3 | 2 | 2 | 3 | 3 | 3 | 3 | 1 | 3 | 35 |
| Ho [74], 2021 | 3 | 3 | 3 | 3 | 3 | 3 | 3 | 3 | 3 | 3 | 3 | 3 | 2 | 38 |
| Werhahn [84],2019 | 2 | 3 | 3 | 3 | 3 | 3 | 3 | 2 | 2 | 3 | 3 | 3 | 2 | 35 |
| Santala [33],2022 | 3 | 3 | 3 | 3 | 3 | 3 | 3 | 3 | 3 | 3 | 3 | 1 | 3 | 37 |
| Ausín [85],2023 | 3 | 3 | 2 | 3 | 2 | 3 | 3 | 3 | 1 | 3 | 3 | 3 | 2 | 34 |
| Kovacs [86],2021 | 3 | 3 | 3 | 2 | 2 | 3 | 3 | 3 | 3 | 2 | 3 | 2 | 3 | 35 |
| Weng [69],2021 | 3 | 3 | 3 | 3 | 3 | 3 | 3 | 2 | 3 | 3 | 3 | 3 | 3 | 38 |
| Campo [75],2022 | 3 | 3 | 3 | 2 | 3 | 3 | 3 | 3 | 3 | 3 | 3 | 2 | 3 | 37 |
| Vaseekaran [76], 2023 | 3 | 3 | 3 | 2 | 2 | 3 | 3 | 3 | 2 | 3 | 3 | 2 | 2 | 34 |
| Lee [94], 2023 | 3 | 3 | 3 | 3 | 3 | 2 | 3 | 3 | 3 | 2 | 3 | 2 | 3 | 36 |
| Peterson [64], 2021 | 2 | 2 | 2 | 2 | 2 | 1 | 2 | 3 | 3 | 1 | 2 | 3 | 2 | 27 |
| Pavic [82],2020 | 3 | 3 | 3 | 3 | 2 | 2 | 3 | 3 | 3 | 2 | 3 | 3 | 3 | 36 |
| Lévi [58],2020 | 3 | 1 | 2 | 2 | 2 | 3 | 3 | 3 | 3 | 3 | 2 | 2 | 0 | 29 |
| Wu [99],2019 | 3 | 3 | 3 | 3 | 2 | 3 | 3 | 3 | 3 | 3 | 3 | 3 | 3 | 38 |
| Ghods [63], 2021 | 3 | 2 | 3 | 2 | 2 | 3 | 3 | 3 | 3 | 1 | 3 | 3 | 2 | 33 |
| Pavic [81], 2020 | 3 | 3 | 3 | 3 | 3 | 3 | 3 | 3 | 3 | 3 | 2 | 3 | 3 | 38 |
| Ayyoubzadeh [97], 2023 | 3 | 3 | 3 | 3 | 2 | 3 | 3 | 3 | 2 | 3 | 3 | 3 | 2 | 36 |
| Filakova [78], 2023 | 3 | 3 | 3 | 3 | 2 | 3 | 3 | 3 | 3 | 3 | 3 | 2 | 3 | 37 |
| Chung [90],2019 | 3 | 3 | 3 | 3 | 3 | 3 | 3 | 3 | 3 | 3 | 3 | 2 | 3 | 38 |
| Ha [100],2022 | 3 | 3 | 3 | 3 | 3 | 3 | 3 | 3 | 3 | 3 | 3 | 3 | 3 | 39 |
| Gao [34], 2023 | 3 | 3 | 3 | 3 | 3 | 3 | 3 | 3 | 3 | 3 | 3 | 3 | 3 | 39 |
| Albani [31], 2019 | 3 | 3 | 3 | 3 | 2 | 3 | 3 | 3 | 3 | 3 | 3 | 2 | 3 | 37 |
| Ruokolainen [24], 2022 | 2 | 3 | 3 | 3 | 3 | 3 | 3 | 3 | 3 | 3 | 3 | 3 | 3 | 38 |
| Lipsmeier [89],2022 | 3 | 3 | 3 | 3 | 3 | 3 | 3 | 3 | 3 | 3 | 3 | 2 | 3 | 38 |
| Gatsios [54],2020 | 3 | 3 | 3 | 3 | 3 | 3 | 3 | 3 | 3 | 3 | 3 | 2 | 3 | 38 |
| Ravichandran [27], 2023 | 2 | 3 | 3 | 3 | 3 | 0 | 3 | 3 | 3 | 0 | 3 | 0 | 2 | 28 |
| Lee [65], 2023 | 3 | 3 | 3 | 2 | 2 | 0 | 2 | 3 | 3 | 0 | 3 | 1 | 2 | 27 |
| Blanc [35],2022 | 3 | 3 | 3 | 2 | 2 | 1 | 3 | 3 | 3 | 0 | 3 | 0 | 0 | 26 |
| Huo [56],2020 | 3 | 3 | 3 | 3 | 2 | 3 | 3 | 3 | 0 | 2 | 3 | 0 | 0 | 28 |
| Sharma [73],2023 | 2 | 3 | 3 | 3 | 2 | 3 | 3 | 3 | 3 | 3 | 3 | 0 | 3 | 34 |
| Sigcha [87],2023 | 3 | 3 | 3 | 3 | 1 | 3 | 3 | 3 | 2 | 3 | 3 | 0 | 3 | 33 |
| Zedda [25],2020 | 2 | 3 | 2 | 2 | 0 | 1 | 2 | 2 | 0 | 0 | 0 | 0 | 0 | 14 |
| Toh [101],2023 | 3 | 3 | 3 | 3 | 3 | 3 | 3 | 3 | 3 | 2 | 3 | 0 | 3 | 35 |
| Marin-pardo [26], 2021 | 2 | 3 | 3 | 3 | 1 | 1 | 3 | 3 | 0 | 0 | 3 | 0 | 3 | 25 |
| Noorian [70],2019 | 1 | 3 | 1 | 2 | 2 | 0 | 2 | 2 | 1 | 0 | 2 | 0 | 0 | 16 |
| Guo [98],2023 | 2 | 3 | 3 | 3 | 3 | 2 | 3 | 3 | 3 | 2 | 3 | 2 | 2 | 34 |
| Darcy [68],2023 | 2 | 3 | 3 | 2 | 2 | 2 | 3 | 3 | 2 | 2 | 3 | 2 | 3 | 32 |
| Chae [91],2020 | 2 | 3 | 3 | 3 | 2 | 2 | 3 | 3 | 3 | 2 | 3 | 1 | 2 | 32 |
| Shivaraja [92],2023 | 3 | 3 | 3 | 3 | 1 | 2 | 3 | 3 | 2 | 2 | 3 | 2 | 2 | 32 |
| Frankel [66],2021 | 2 | 3 | 3 | 3 | 3 | 3 | 3 | 3 | 3 | 3 | 3 | 1 | 2 | 35 |
| Scholten [28], 2022 | 2 | 3 | 3 | 2 | 2 | 1 | 2 | 2 | 2 | 1 | 2 | 0 | 2 | 24 |
| Waddell [72], 2023 | 2 | 3 | 3 | 3 | 2 | 2 | 3 | 3 | 3 | 2 | 3 | 1 | 3 | 33 |
| Ramesh [96], 2021. | 3 | 3 | 2 | 3 | 2 | 2 | 3 | 3 | 1 | 2 | 3 | 1 | 3 | 31 |
| Boscari [53],2021 | 2 | 2 | 3 | 3 | 2 | 2 | 3 | 3 | 2 | 2 | 3 | 0 | 2 | 29 |
| Bergenstal [32], 2021 | 3 | 2 | 3 | 2 | 1 | 2 | 2 | 3 | 2 | 2 | 2 | 2 | 2 | 28 |
| Cappon [55],2022 | 2 | 2 | 2 | 2 | 1 | 2 | 3 | 3 | 1 | 0 | 0 | 2 | 2 | 22 |
| Notemi [83],2022 | 2 | 3 | 3 | 2 | 2 | 2 | 3 | 3 | 2 | 3 | 3 | 2 | 2 | 32 |
| Forlenza [60], 2019 | 2 | 3 | 3 | 3 | 3 | 3 | 3 | 3 | 3 | 2 | 3 | 2 | 3 | 36 |
| Beach [59],2021 | 3 | 3 | 3 | 3 | 2 | 2 | 3 | 3 | 3 | 2 | 3 | 1 | 3 | 34 |
| Kytö [80],2023 | 3 | 3 | 3 | 3 | 2 | 3 | 3 | 3 | 2 | 2 | 3 | 1 | 2 | 33 |
| Knijffa [29], 2022 | 2 | 3 | 3 | 3 | 2 | 2 | 3 | 3 | 2 | 2 | 3 | 1 | 3 | 32 |
| Lee [95],2019 | 2 | 3 | 3 | 2 | 2 | 3 | 3 | 3 | 3 | 3 | 3 | 1 | 3 | 34 |
| Bui [61],2020 | 2 | 3 | 3 | 3 | 2 | 2 | 2 | 3 | 3 | 1 | 3 | 2 | 2 | 31 |
| Radogna [52],2020 | 3 | 3 | 3 | 3 | 2 | 3 | 3 | 3 | 1 | 1 | 2 | 2 | 2 | 31 |
| Althobiani [57],2023 | 2 | 3 | 2 | 3 | 2 | 3 | 3 | 3 | 3 | 3 | 3 | 3 | 3 | 36 |
| Morales-botello [88], 2021 | 2 | 2 | 1 | 2 | 0 | 3 | 3 | 3 | 0 | 3 | 3 | 2 | 2 | 26 |

**Note:** 0 = not at all, 1 = very slightly, 2 = moderately, 3 = complete.

**QuADS criteria:**

**C1**: Theoretical or conceptual underpinning to the research

**C2**: Statement of research aim/s

**C3**: Clear description of research setting and target population

**C4**: The study design is appropriate to address the stated research aim/s

**C5**: Appropriate sampling to address the research aim/s

**C6**: Rationale for choice of data collection tool/s

**C7**: The format and content of data collection tool is appropriate to address the stated research aim/s

**C8**: Description of data collection procedure

**C9**: Recruitment data provided

**C10**: Justification for analytic method selected

**C11**: The method of analysis was appropriate to answer the research aim/s

**C12**: Evidence that the research stakeholders have been considered in research design or conduct

**C13**: Strengths and limitations critically discussed
